# Draft Genome Sequence of the Rice Endophyte *Burkholderia kururiensis* M130

**DOI:** 10.1128/genomeA.00225-12

**Published:** 2013-04-04

**Authors:** Bruna Gonçalves Coutinho, Daniel Passos da Silva, José Osvaldo Previato, Lucia Mendonça-Previato, Vittorio Venturi

**Affiliations:** International Centre for Genetic Engineering & Biotechnology, Trieste, Italya;; The Capes Foundation, Ministry of Education of Brazil, Brasilia, DF, Brazilb;; Universidade Federal do Rio de Janeiro, Centro de Ciências da Saúde, Instituto de Biofísica Carlos Chagas Filho-Ilha da Cidade Universitária, Rio de Janeiro, RJ, Brazilc

## Abstract

*Burkholderia kururiensis* M130 is one of the few characterized rice endophytes and was isolated from surface-sterilized rice roots. This bacterium shows strong growth-promoting effects, being able to increase rice yields. Here we present its draft genome sequence, which contains important traits for endophytic life and plant growth promotion.

## GENOME ANNOUNCEMENT

Rice is the basic food for a large proportion of the world’s population (1). The production of this crop must increase by ~1% annually to meet the growing demand for food resulting from population growth and economic development (2). Most of this increase must derive from improvement of the yields on existing croplands to avoid destruction of natural ecosystems and loss of biodiversity (3, 4). An environmentally friendly strategy to improve rice production is the use of nitrogen-fixing endophytic bacteria (5).

The sequenced bacterium, *Burkholderia kururiensis* M130, formerly named “*Burkholderia brasilensis*,” was isolated from surface-sterilized rice roots in Brazil; however, strains from the same species were also recovered from banana, pineapple, sugarcane, tobacco, and cassava (6–9). Further taxonomical analysis, which included 16S rRNA, *nifH*, *glnB*, and other markers, determined that “*B. brasilensis*” was actually a *B. kururiensis* strain (10, 11). Strains from this species are diazotrophs, increasing the nitrogen availability to the plant and consequently promoting enhancement in growth and yield (6, 12). In agreement with this, the inoculation of *B. kururiensis* M130 in rice seeds showed that from 28% to 31% of the total N accumulated by the plant had been fixed by the bacteria (12). Moreover, several experiments demonstrated that the inoculation of this strain in different rice cultivars promoted an increase of more than 30% in rice yields (12).

Genome sequencing was performed using a combination of Illumina (13) and 454 (14) technologies. An Illumina GAII shotgun library (3,320,000 reads totaling 332.0 Mb) and a paired-end 454 GS-FLX library (445,966 reads totaling 163.7 Mb) were generated and sequenced. Altogether, 3,961,173 pairs of reads were obtained, with an ~60-fold coverage of the ~7.1-Mb genome. The *de novo* assembly using MIRA (Mimicking Intelligent Read Assembly) version 3.4.0 (15) followed by a manual curation yielded 83 contigs organized in 9 scaffolds. The longest scaffold obtained was 1,922 kb long. The genome of *B. kururiensis* M130 presents a G+C content of 63% and, according to the automated annotation done using RAST (16), contains a total of 6,579 predicted protein-coding genes, from which 1,795 (27.3%) were annotated as encoding hypothetical proteins. A total of 63 RNA coding sequences were also identified in the RAST annotation.

The *B*. *kururiensis* M130 genome presents several genes related to plant growth promotion, including the *accD* gene encoding 1-aminocyclopropane-1-carboxylate deaminase, genes for the production of indole-3-acetic acid, and the *nif* gene cluster. The genome apparently does not possess genes for the production of antibiotics, and it presents one *N*-acylhomoserine lactone quorum-sensing system. The M130 genome shows a range of detoxification mechanisms, including for the degradation of organic substances, heavy metal efflux systems, and various enzymes necessary to cope with oxidative stress. In the present study, we determined the draft genome information of a strain of *B. kururiensis* with great biotechnological potential. To our knowledge, this is the first sequence report of this species.

### Nucleotide sequence accession numbers. 

This whole-genome shotgun project has been deposited at DDBJ/EMBL/GenBank under the accession number ANSK00000000. The version described in this paper is the first version, ANSK01000000.
